# Olive oil intake and cancer risk: A systematic review and meta-analysis

**DOI:** 10.1371/journal.pone.0261649

**Published:** 2022-01-11

**Authors:** Christos Markellos, Maria-Eleni Ourailidou, Maria Gavriatopoulou, Panagiotis Halvatsiotis, Theodoros N. Sergentanis, Theodora Psaltopoulou

**Affiliations:** 1 Department of Clinical Therapeutics, “Alexandra” Hospital, School of Medicine, University of Athens, Athens, Greece; 2 Second Department of Internal Medicine, Athens School of Medicine, Attikon University Hospital, Athens, Greece; University of Palermo, ITALY

## Abstract

**Background:**

Research evidence has established the beneficial effects of diet in cancer prevention; various epidemiological studies have suggested that olive oil component could play a role in decreasing cancer risk. This systematic review and meta-analysis aims to investigate the association between olive oil consumption, cancer risk and prognosis.

**Methods:**

A systematic search was conducted in PubMed, EMBASE and Google Scholar databases (end-of-search: May 10, 2020). Pooled relative risk (RR) and 95% confidence intervals (95% CIs) were estimated with random-effects (DerSimonian-Laird) models. Subgroup analyses, sensitivity analyses and meta-regression analysis were also performed.

**Results:**

45 studies were included in the meta-analysis; 37 were case-control (17,369 cases and 28,294 controls) and 8 were cohort studies (12,461 incident cases in a total cohort of 929,771 subjects). Highest olive oil consumption was associated with 31% lower likelihood of any cancer (pooled RR = 0.69, 95%CI: 0.62–0.77), breast (RR = 0.67, 95%CI: 0.52–0.86), gastrointestinal (RR = 0.77, 95%CI: 0.66–0.89), upper aerodigestive (RR = 0.74, 95%CI: 0.60–0.91) and urinary tract cancer (RR = 0.46, 95%CI: 0.29–0.72). Significant overall effects spanned both Mediterranean and non-Mediterranean participants, studies presenting a multivariate and a univariate analysis and all subgroups by study quality.

**Conclusions:**

Olive oil consumption seems to exert beneficial actions in terms of cancer prevention. Additional prospective cohort studies on various cancer types and survivors, as well as large randomized trials, seem desirable.

## 1. Introduction

Cancer is accountable for an estimated 9.6 million deaths in 2018, being the second leading cause of death globally, only after cardiovascular diseases [1]. The economic burden of cancer on patients and healthcare systems is substantial and increasing, with a worldwide annual economic toll in 2010 estimated at approximately US$ 1.16 trillion [1]. The role of diet as an important, potentially modifiable factor in cancer prevention has been highlighted [2–4]. According to the World Cancer Research Fund (WCRF), 40% of cancer cases can be prevented by appropriate diet, nutrition and physical activity [5]. However, the attributable detrimental impact of diet on cancer incidence seems to be increasing nowadays [6].

Accumulating evidence has pointed to a reduction in the risk of various types of cancer in populations of the Mediterranean basin, largely due to high consumption of olive oil as the main vegetable fat, plant-based foods and fish, as well as to a moderate consumption of white meat, eggs, dairy products and alcohol [7–13]. Olive oil (*Olea europaea*, *Oleaceae*) is a traditional staple food for Mediterranean people and a fundamental component of the Mediterranean diet, used for both dressing and cooking. It has the highest ratio of monounsaturated to polyunsaturated fatty acids among vegetable oils. Its favorable effects have been attributed to the abundance of valuable nutrients, such as antioxidant phenolic compounds (i.e., hydroxytyrosol and oleuropein), vitamins, lignans, squalene and terpenoids [14–18].

Recent bibliography from *in vitro* and animal nutrigenomics studies suggests that olive oil components act on receptors, signaling kinases and transcription factors associated with cellular stress and inflammation, lipoprotein metabolism and damage, endothelial function and, in general, with pathways responsible for cell cycle regulation and metabolism, exerting a protective role on malignancy development [14, 16, 19–23]. To date, the relationship between olive oil consumption and cancer risk in humans has been studied in epidemiological studies, most taking place around the Mediterranean region, where populations consume it in large quantities, reporting equivocal associations [13, 24–29]. In our previous meta-analysis of 19 case-control studies, conducted nearly 10 years ago, we observed a significant inverse relationship between olive oil intake and overall cancer risk [30]; nevertheless, a considerable amount of evidence has been accumulated thereafter, allowing further insight in overall and site-specific associations.

For the scope of the present study, we conducted a systematic review and meta-analysis of all the available epidemiological studies that have assessed the association between olive oil consumption and cancer risk or prognosis, aiming, ultimately, at establishing the role of olive oil intake in cancer prevention and survival.

## 2. Material and methods

### 2.1 Search strategy and eligibility of studies

The present systematic review and meta-analysis was performed following the Preferred Reporting Items for Systematic Reviews and Meta-Analyses (PRISMA) guidelines [31]; the PRISMA Checklist is presented in S1 Table in S1 File. The study protocol was discussed and agreed upon in advance by all authors. A systematic search was conducted in PubMed, EMBASE and Google Scholar databases (end-of-search: May 10, 2020). In PubMed and EMBASE, the following search algorithm was used: olive AND oil AND cancer. As far as publication language is concerned, no restriction was implemented. Reference lists of reviews and eligible articles were systematically searched for relevant articles in a “snowball” procedure. The search in Google Scholar was performed using the keywords “olive oil” and cancer; articles including these words were sorted by best matching and the first 1000 hits were screened.

Eligible articles included randomized controlled trials, case-control, cohort and cross-sectional studies investigating the association between higher *versus* lower intake of olive oil with cancer risk (incidence; mortality) and prognosis. Case series and case reports, reviews, *in vitro* and animal studies were not included in this meta-analysis. In case of overlapping study populations, only the larger study was included. The selection of studies was performed by two reviewers (CM, MO) working independently and any disagreements were resolved following consultation with a senior author (TNS) and team consensus.

### 2.2 Data abstraction and effect estimates

The abstraction of data encompassed general information (first author’s name, study year), study characteristics [study design, time period, geographical region, number of cases and controls (for case–control studies), matching factors (for case–control studies), follow-up period, cohort size and incident cases (for cohort studies)], definition of olive oil intake, categorization of exposure, features of ascertainment for exposure and characteristics of participants [inclusion and exclusion criteria, age of participants (range, mean), percentage of males], as well as adjusting factors regarding multivariate analyses. If the required data for the meta-analysis were not readily available in the published article, the corresponding authors were contacted twice (a reminder e-mail was sent seven days after the first e-mail). Data were independently extracted, analyzed and recorded in a predeveloped data extraction sheet by two reviewers (CM and MO). Final decision was reached after consultation with a senior author (TNS) and team consensus.

The maximally adjusted effect estimates i.e., odds ratios (ORs) for case–control studies, relative risks (RRs) or hazard ratios (HRs) in case of randomized controlled trials and non-randomized cohort studies with their confidence intervals (CIs) were extracted from each study by category of olive oil intake. In case the aforementioned information was not available, crude effect estimates and 95% CIs were calculated by means of 2x2 tables presented in the articles.

### 2.3 Statistical analyses

Statistical analyses included pooling of studies as well as *post hoc* meta-regression and sensitivity analyses. Statistical synthesis was performed in case of two or more eligible study arms. Random-effects (DerSimonian–Laird) models were appropriately used to calculate pooled effect estimates. The category of highest olive oil intake was compared with the one corresponding to the lowest consumption. Between-study heterogeneity was assessed by estimating Q-test and I^2^ [32]. Separate analyses were performed based on cancer site, study design, geographic region (grouped as Mediterranean, mixed Mediterranean and non-Mediterranean studies), degree of adjustment and overall study quality. A *post hoc* subgroup analysis was performed in studies examining olive oil consumption within or outside the context of adherence to Mediterranean diet. *Post hoc* sensitivity analyses were performed excluding the following effect estimates; first those pertaining to cancer mortality (and not purely incidence) and second, in the site-specific analyses, a study incorporating in the pool of upper aerodigestive cancer also esophageal cancer cases [33]. Meta-regression analysis was performed in cases of 10 or more pooled study arms [32] and aimed to assess whether gender (expressed as percentage of males in the individual studies), age (expressed as the mean age in the individual studies) and publication year modified the association between olive oil consumption and cancer risk. Statistical analysis and meta-regression analysis were performed using STATA/SE version 13 (Stata Corp, College Station, TX, USA).

### 2.4 Assessment of study quality and publication bias

As far as the risk of bias is concerned, the Newcastle-Ottawa Quality scale [34] was used to evaluate the quality of the included non-randomized studies. Regarding the items assessing the completeness (adequacy) of follow-up of cohorts and whether the follow-up period was enough for outcomes to occur, the cut-off values were set *a priori* at 85% response rate and 5 years, respectively. Study quality was considered “low” when the Newcastle-Ottawa score (NOS) ranged between 1–3, “intermediate” for studies with NOS between 4–6 and “high” for those with a score between 7–9. Two independently working reviewers (CM, MO) rated the studies and, in case of disagreement, final decision was reached after consultation with a senior author (TNS) and team consensus.

Publication bias was evaluated in the analyses that included 10 or more study arms [32]; Egger’s statistical test [35] was implemented as well as a visual inspection of the funnel plot. For the interpretation of Egger’s test, statistical significance was defined as p<0.1. The evaluation of publication bias was performed using STATA/SE version 13 (Stata Corp, College Station, TX, USA).

## 3. Results

### 3.1 Description of eligible studies

A total of 3813 records were identified (998 from Pubmed, 1815 from EMBASE, 1000 from Google Scholar) using the search algorithm. After duplicates were removed, 2413 abstracts were screened; all details pertaining to the successive steps for the selection of eligible studies are provided in the supplemental material (Supplemental Results, S1 Fig and S2 Table in S1 File).

**Fig 1:**
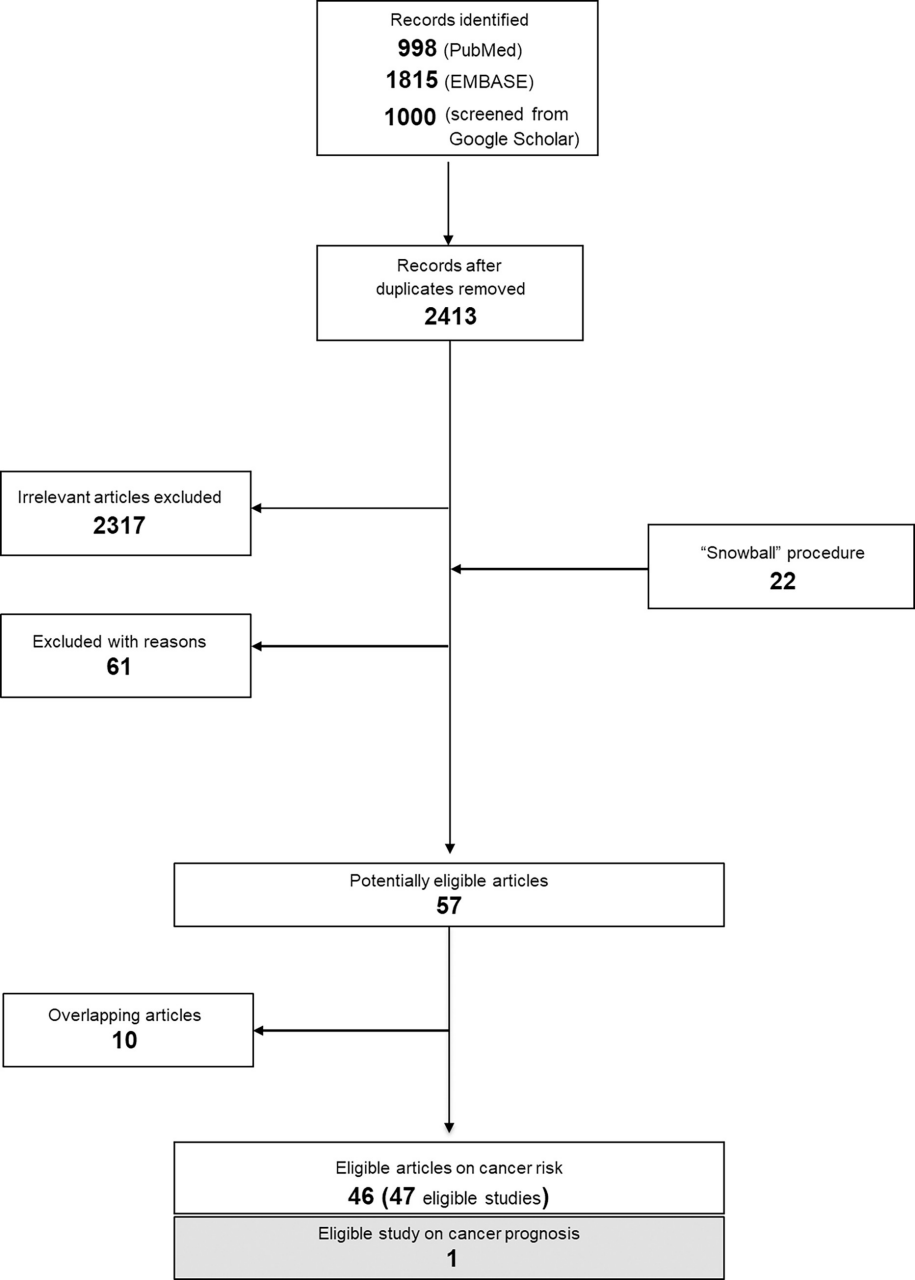
Flow chart presenting the successive steps in the selection of eligible studies.

47 articles that resulted in 48 studies and explored the association between olive oil intake and cancer risk [33, 36–80] or prognosis [81] were finally considered eligible (Fig 1); 38 were case-control (18,303 cases and 29,109 controls, S3 Table in S1 File) [33, 36–54, 56, 57, 60–70, 73, 76, 78, 79] and 10 were cohort studies (13,448 incident cases among a total cohort size equal to 955,609 subjects, S4 and S5 Tables in S1 File) [55, 58, 59, 71, 72, 74, 75, 77, 80, 81]. The study by Toledo *et al*. [59] was grouped with cohort studies; despite a randomized design, the comparison included in our study stemmed from pooled assessment of all three trial groups in a per protocol analysis. Among the 38 case-control studies, 28 included population-based controls, whereas 10 included hospital-based controls.

Overall, 15 studies examined the association between olive oil consumption and the risk of breast cancer [38–40, 42, 50, 51, 54, 59, 63, 66–68, 75, 78, 80], one article reported on ovarian cancer [47], one on lung [77], three on gastric [37, 61, 72], six on colorectal [41, 60, 62, 64, 73, 74], among which two provided data on colon and rectal cancers as well [41, 64], one on both gastric and colorectal [57], one on pancreatic [43], nine on cancers of the upper aerodigestive tract [33, 45, 46, 48, 52, 56, 65, 69, 70] and six on urinary tract cancers (bladder [53], prostate [44, 49, 76, 79], any site [36]). Two studies referred to the relationship with overall cancer mortality [55, 58] and one with overall cancer risk [71].

Two studies investigated only the effect of incremental olive oil consumption [54, 71] and, thus, were included in the qualitative synthesis. This was also the case for the study by Crosignani *et al*., as it was the only one to investigate the effects of olive oil intake on survival (in that instance, of male laryngeal cancer patients) [81]. The evaluation of study quality is presented in S6 and S7 Tables in S1 File.

### 3.2 Meta-analysis

**Fig 2:**
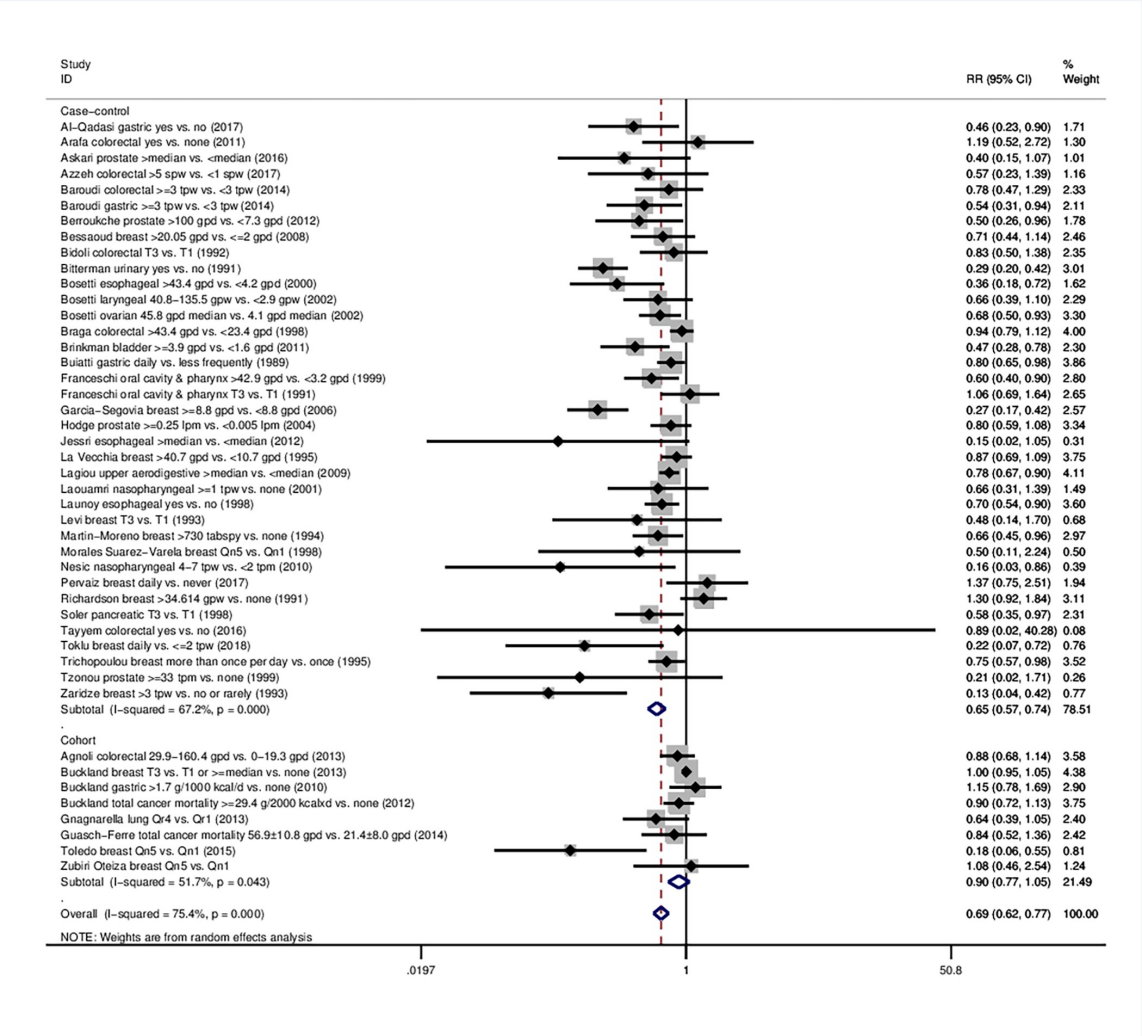
Forest plot describing the association between high olive oil consumption and risk for cancer. Apart from the overall analysis, the subanalyses on case-control (upper panels) and cohort studies (lower panels) are presented.

45 eligible studies were included in the overall meta-analysis; 37 were case-control (17,369 cases and 28,294 controls) and 8 were cohort studies (12,461 incident cases in a total cohort of 929,771 subjects) (Fig 2, Table 1). The combined effect of the highest stratum of olive oil intake compared with the lowest was statistically significant. More specifically, highest olive oil consumption was associated with 31% lower likelihood of developing any type of cancer (RR = 0.69, 95% CI: 0.62–0.77).

**Table 1:** Results of the meta-analyses examining the association between olive oil consumption and cancer risk; subgroup analyses by cancer site, study design and geographic region are presented. Bold cells denote statistically significant associations.

	“Highest vs. lowest” comparison		
	n^§^	RR (95%CI:)	Heterogeneity I^2^, p
**Analysis on overall cancer**			
Overall analysis	45	**0.69 (0.62–0.77)**	75.4%, <0.001
Subgroups by study design			
*Case-control studies*	37	**0.65 (0.57–0.74)**	67.2%, <0.001
*Cohort studies*	8	0.90 (0.77–1.05)	51.7%, 0.043
Subgroups by geographic region			
*Mediterranean*	30	**0.69 (0.60–0.79)**	70.2%, <0.001
*Mixed Mediterranean*	4	0.90 (0.74–1.10)	76.3%, 0.005
*Non-Mediterranean*	11	**0.49 (0.34–0.71)**	48.5%, 0.035
**Analysis on breast cancer**			
Overall analysis	14	**0.67 (0.52–0.86)**	82.5%, <0.001
Subgroups by study design			
*Case-control studies*	11	**0.63 (0.45–0.87)**	79.5%, <0.001
*Cohort studies*	3	0.67 (0.29–1.56)	77.6%, 0.011
Subgroups by geographic region			
*Mediterranean*	11	**0.67 (0.49–0.92)**	78.5%, <0.001
*Mixed Mediterranean*	1	1.00 (0.95–1.05)	NC
*Non-Mediterranean*	2	**0.25 (0.07–0.89)**	55.9%, 0.132
**Analysis on gastrointestinal cancer**			
Overall analysis	15	**0.77 (0.66–0.89)**	40.6%, 0.052
Subgroups by study design			
*Case-control studies*	13	**0.72 (0.61–0.85)**	38.5%, 0.077
*Cohort studies*	2	0.97 (0.75–1.24)	21.4%, 0.259
Subgroups by geographic region			
*Mediterranean*	9	**0.77 (0.67–0.88)**	39.9%, 0.101
*Mixed Mediterranean*	1	1.15 (0.78–1.69)	NC
*Non-Mediterranean*	5	0.60 (0.35–1.03)	24.1%, 0.261
Subgroups by site			
*Colorectal*	7	0.90 (0.79–1.03)	0.0%, 0.906
*Esophageal*	3	**0.47 (0.24–0.93)**	61.5%, 0.074
*Gastric*	4	0.75 (0.53–1.05)	62.0%, 0.048
*Pancreatic*	1	**0.58 (0.35–0.97)**	NC
**Analysis on upper aerodigestive cancer**			
Overall analysis	6	**0.74 (0.60–0.91)**	32.7%, 0.191
Subgroups by study design			
*Case-control studies*	6	**0.74 (0.60–0.91)**	32.7%, 0.191
*Cohort studies*	0	No studies	
Subgroups by geographic region			
*Mediterranean*	3	0.76 (0.51–1.13)	45.8%, 0.158
*Mixed Mediterranean*	2	**0.77 (0.67–0.89)**	0.0%, 0.540
*Non-Mediterranean*	1	**0.16 (0.03–0.86)**	NC
Subgroups by site			
*Laryngeal*	1	0.66 (0.39–1.10)	NC
*Nasopharyngeal*	2	0.40 (0.10–1.54)	56.9%, 0.128
*Oral/pharyngeal*	2	0.79 (0.45–1.39)	71.6%, 0.061
*Upper aerodigestive tract*, *any site*	1	**0.78 (0.67–0.90)**	NC
**Analysis on urinary cancer**			
Overall analysis	6	**0.46 (0.29–0.72)**	72.9%, 0.002
Subgroups by study design			
*Case-control studies*	6	**0.46 (0.29–0.72)**	72.9%, 0.002
*Cohort studies*	0	No studies	
Subgroups by geographic region			
*Mediterranean*	3	**0.33 (0.23–0.48)**	8.6%, 0.335
*Mixed Mediterranean*	0	No studies	
*Non-Mediterranean*	3	**0.60 (0.38–0.93)**	52.5%, 0.122
Subgroups by site			
*Prostate*	4	**0.61 (0.40–0.92)**	30%, 0.232
*Bladder*	1	**0.47 (0.28–0.78)**	NC
*Urinary tract*, *any site*	1	**0.29 (0.20–0.42)**	NC

^§^number of study arms; NC: not calculable; NOS: Newcastle-Ottawa scale.

The protective effect of high olive oil consumption in terms of cancer risk was also reflected within the subset of case-control studies (37 study arms, RR = 0.65, 95%CI: 0.57–0.74); a non-significant trend was observed in cohort studies (8 study arms, RR = 0.90, 95%CI: 0.77–1.05) (Table 1, Fig 2). Regarding geographic region, both Mediterranean and non-Mediterranean participants that reported higher olive oil intake were significantly less likely to develop any type of cancer (RR = 0.69, 95%CI: 0.60–0.79 and RR = 0.49, 95%CI: 0.34–0.71 respectively) (Table 1, S2 Fig in S1 File). The protective association spanned studies presenting a multivariate analysis (32 study arms, RR = 0.72, 95%CI: 0.65–0.81) and a univariate analysis (13 study arms, RR = 0.57, 95%CI: 0.40–0.82) (S8 Table, S3 Fig in S1 File). Similarly, a highly significant association was noted in all subgroups by study quality (low; RR = 0.29, 95%CI: 0.20–0.42, intermediate; RR = 0.69, 95%CI: 0.53–0.90, high; RR = 0.72, 95%CI: 0.64–0.81) (S8 Table, S4 Fig in S1 File). A *post hoc* subgroup analysis showed similar results in studies examining olive oil consumption in the context of adherence to Mediterranean diet (nine study arms, RR = 0.74, 95%CI: 0.56–0.96) and outside that context (36 study arms, RR = 0.68, 95%CI: 0.60–0.77). The results persisted in the *post hoc* sensitivity analysis excluding studies on cancer mortality (RR = 0.68, 95%CI: 0.60–0.76) (S5 Fig in S1 File).

### 3.3 Breast cancer

**Fig 3:**
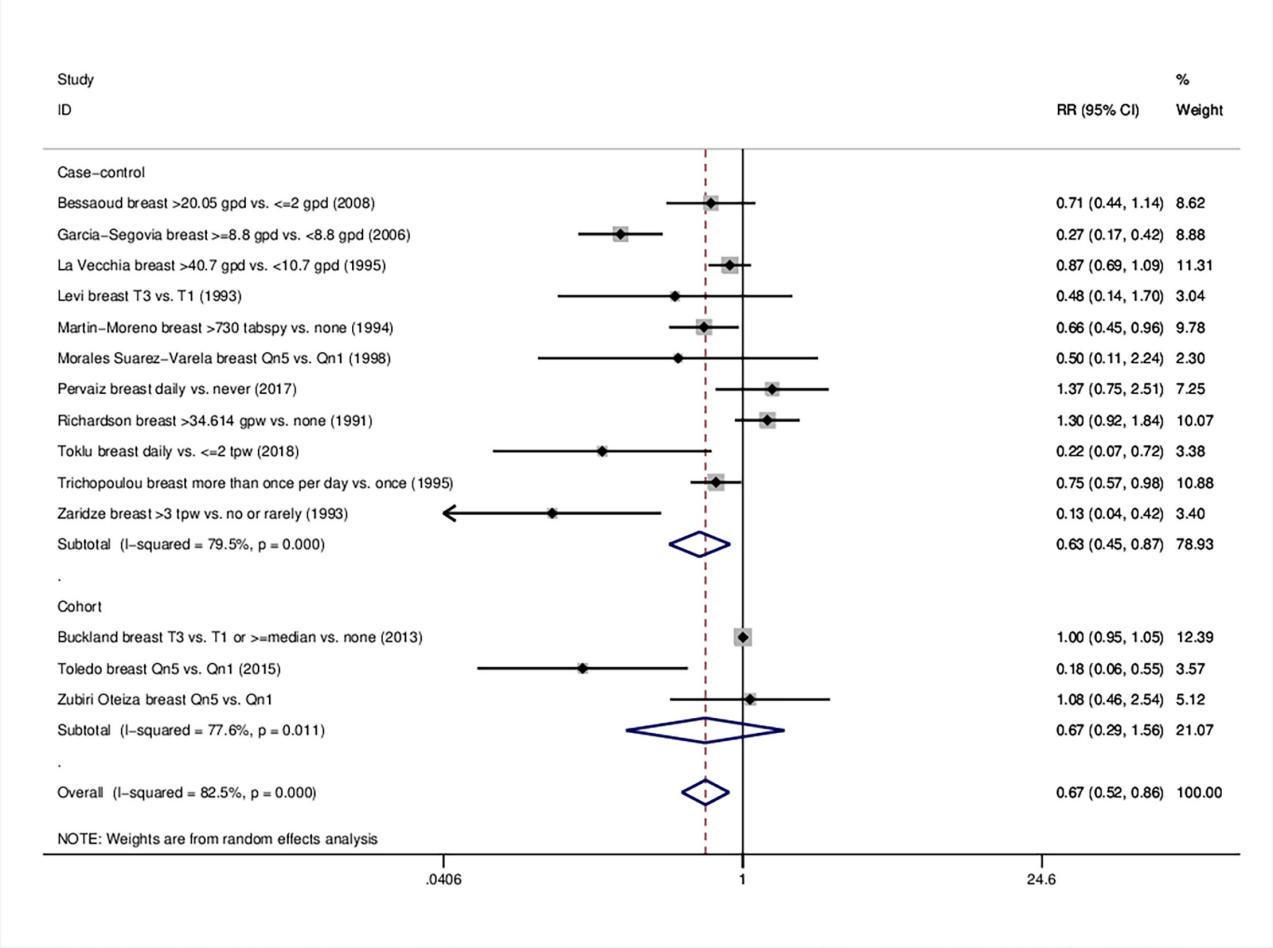
Forest plot describing the association between high olive oil consumption and risk for breast cancer. Apart from the overall analysis, the subanalyses on study design are presented.

As far as breast cancer is concerned, pooling of 14 study arms resulted in a protective association (RR = 0.67, 95%CI 0.52–0.86). Similarly to the analysis on overall cancer, the beneficial effect was reproducible in case-control (RR = 0.63, 95%CI: 0.45–0.87) but not in cohort studies (Table 1, Fig 3). Compared with low intake, high olive oil consumption was linked to a reduced breast cancer risk in Mediterranean (RR = 0.67, 95%CI: 0.49–0.92) and non-Mediterranean populations (RR = 0.25, 95%CI: 0.07–0.89). The single mixed population study reported a null effect (Table 1, S6 Fig in S1 File). The decreased risk was consistent in both adjusted and unadjusted effect estimates (RR = 0.71, 95%CI: 0.55–0.92 and RR = 0.36, 95%CI: 0.17–0.75 respectively) (S8 Table in S1 File, S7 Fig in S1 File). Studies of intermediate quality were associated with lower odds of developing breast cancer (RR = 0.34, 95%CI: 0.17–0.70), whereas studies of high quality the results were only marginal (RR = 0.80, 95%CI: 0.62–1.02) (S8 Table in S1 File, S8 Fig in S1 File).

### 3.4 Gastrointestinal cancer

**Fig 4:**
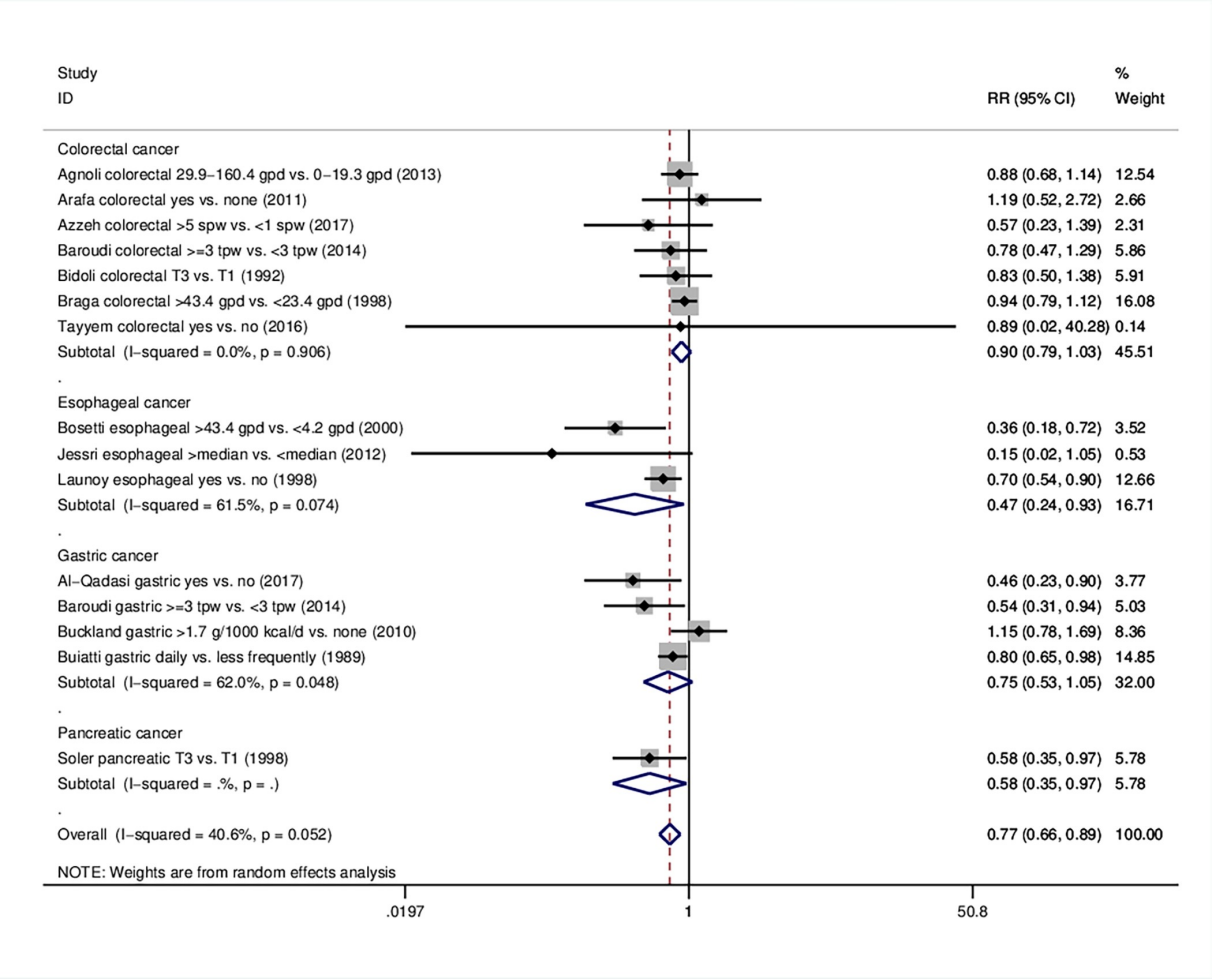
Forest plot describing the association between high olive oil consumption and risk for gastrointestinal cancer. Apart from the overall analysis, the subanalyses per tumor site are presented.

The risk for gastrointestinal cancer was found to be 23% lower for those who consumed the highest amounts of olive oil (RR = 0.77, 95%CI: 0.66–0.89). When we proceeded with subanalyses per tumor site, an inverse relationship was found between olive oil intake and risk for esophageal (RR = 0.47 95%CI: 0.24–0.93) and pancreatic cancer (RR = 0.58, 95%CI: 0.35–0.97) (Table 1, Fig 4); no significance was reached in the site-specific analysis on gastric (RR = 0.75, 95%CI: 0.53–1.05, four studies) and colorectal cancer (RR = 0.90, 95%CI: 0.79–1.03, seven studies). Subgroups that reached significant effects included case-control studies (RR = 0.72, 95%CI: 0.61–0.85), studies within the Mediterranean area (RR = 0.77, 95%CI: 0.67–0.88), multivariate analyses (RR = 0.76, 95%CI: 0.63–0.90) and high quality studies (RR = 0.73, 95%CI: 0.62–0.86). S9-S12 Figs in S1 File portray the results on gastrointestinal cancer as a whole. Information on further subgroups per individual cancer type is illustrated in S9 Table and S13-S26 Figs in S1 File.

### 3.5 Upper aerodigestive cancers

**Fig 5:**
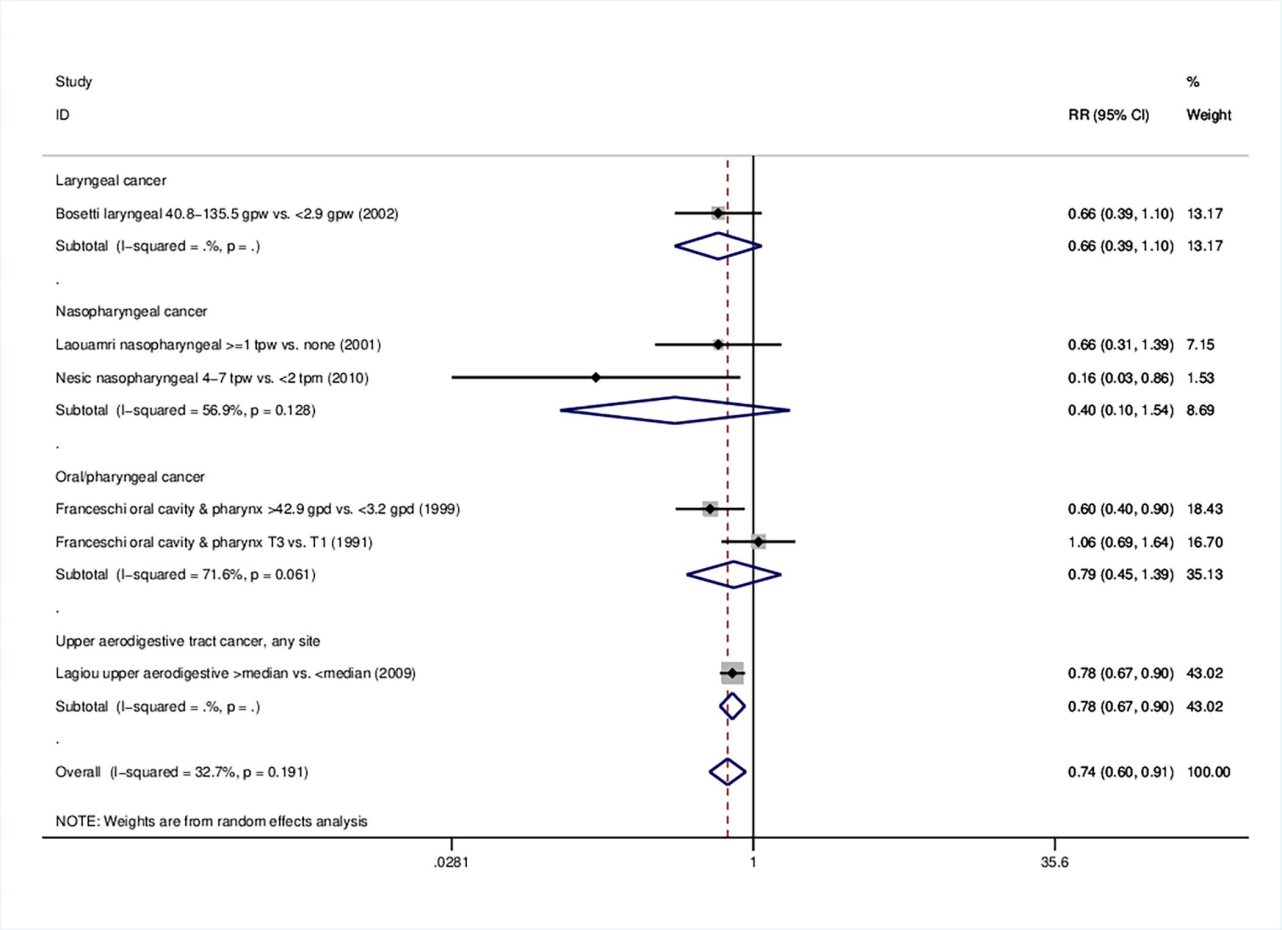
Forest plot describing the association between high olive oil consumption and risk for upper aerodigestive tract cancer. Apart from the overall analysis, the subanalyses per tumor site are presented.

In the case of upper aerodigestive tract cancers, favorable effects of higher olive oil consumption were found (RR = 0.74, 95%CI: 0.60–0.91) (Table 1); subgroup analyses by site did not reach significance, including one or, at most, two studies (Fig 5). At the sensitivity analysis excluding the study whose cases were admixed with esophageal cancer cases [33], the pooled estimate remained significant (RR = 0.69, 95%CI: 0.49–0.98) (S27 Fig in S1 File). Results remained significant for case-control studies (RR = 0.74, 95%CI: 0.60–0.91), studies of mixed (RR = 0.77, 95%CI: 0.67–0.89) and non-Mediterranean origin (RR = 0.16, 95%CI: 0.03–0.86), multivariate analyses (RR = 0.75, 95%CI: 0.66–0.86) and studies of high quality (RR = 0.68, 95%CI: 0.52–0.89) (S8 Table in S1 File, S28-S31 Figs in S1 File).

### 3.6 Urinary tract cancers

**Fig 6:**
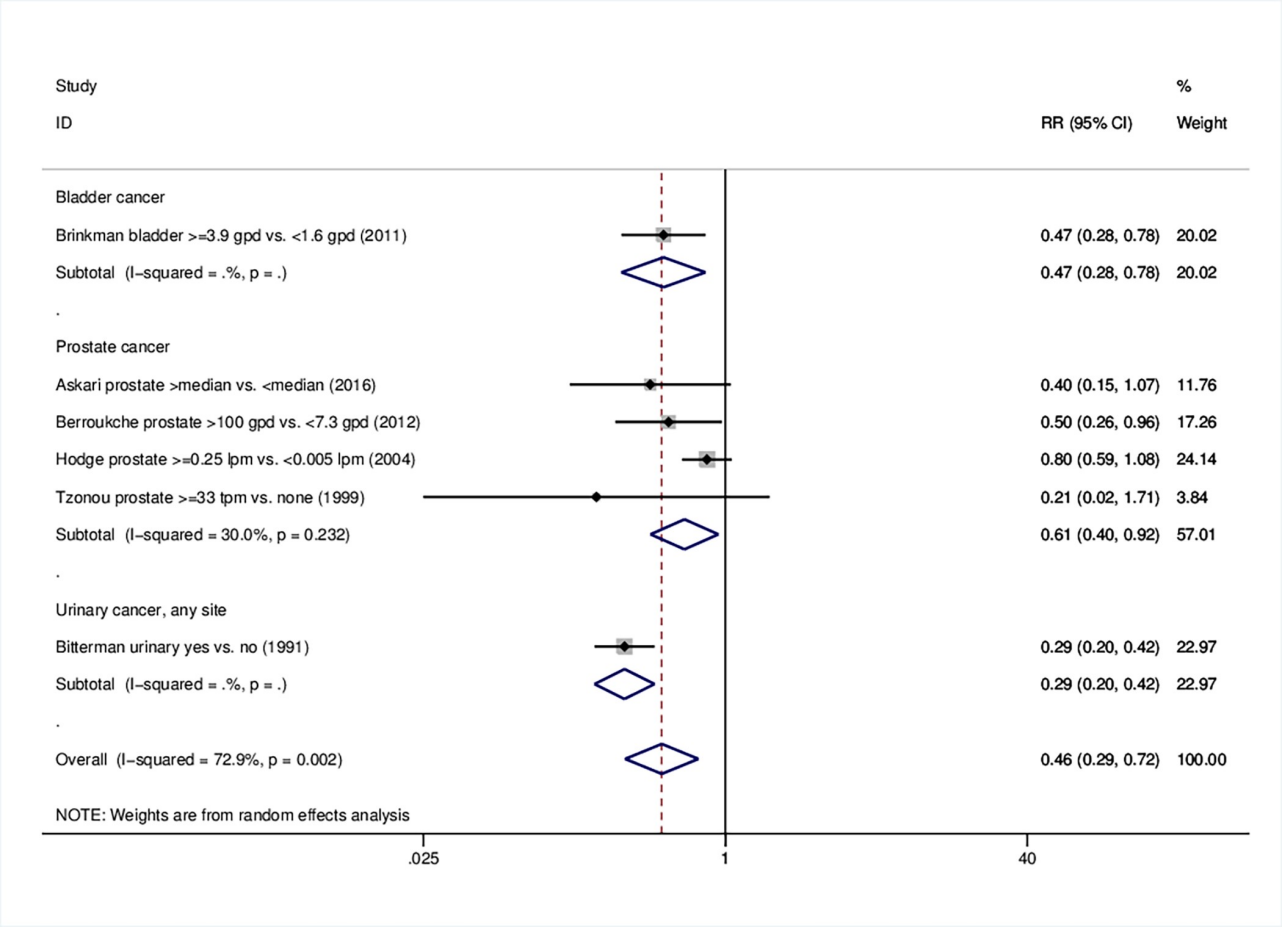
Forest plot describing the association between high olive oil consumption and risk for urinary tract cancer. Apart from the overall analysis, the subanalyses per tumor site are presented.

Remarkably, pooled analysis on urinary tract cancers indicated a relative risk of 0.46 (95%CI: 0.29–0.72) (Table 1); examination of all relevant sites indicated an inverse association with olive oil intake (prostate; RR = 0.61, 95%CI: 0.40–0.92), bladder; RR = 0.47, 95%CI: 0.28–0.78; urinary tract, any site; RR = 0.29, 95%CI: 0.20–0.42) (Table 1, Fig 6). All studies were conducted using a case-control design; the strong protective effects were reproducible on any origin and degree of adjustment, as well as in lowest and highest quality scores (RR = 0.28, 95%CI: 0.20–0.42 and RR = 0.46, 95%CI: 0.32–0.66, respectively) (S8 Table in S1 File, S32-S35 Figs in S1 File). Forest plots and meta-analysis data on prostate study arms are presented in S7 Table and S36-S39 Figs in S1 File.

### 3.7 Qualitative synthesis on studies not included in the meta-analysis

The relevant passage is presented in the S1 File and S10 Table in S1 File.

### 3.8 Meta-regression analysis

Table 2 presents the results of meta-regression analyses. A null effect on overall and gastrointestinal cancer risk was observed when mean age was studied. Similarly, publication year did not modify the decrease in overall, breast and gastrointestinal cancer incidence by olive oil consumption. The protective effects mediated by high olive oil intake in terms of gastrointestinal cancer risk seemed marginally more pronounced among males (exponentiated coefficient = 0.94, 95%CI: 0.89–1.00) (S40 Fig in S1 File). On the other hand, gender did not modify the association with overall cancer risk.

**Table 2:** Meta-regression analysis examining the role of potential modifiers in the association between cancer risk and olive oil consumption.

Variables	Category or increment	Overall cancer			Breast cancer			Gastrointestinal cancer		
		n^§^	Exponentiated coefficient (95%CI)	P	n^§^	Exponentiated coefficient (95%CI)	p	n^§^	Exponentiated coefficient (95%CI)	p
Percentage of males	10% increase	44	0.99 (0.95–1.03)	0.540	-	N/A		15	**0.94 (0.89–1.00)**	**0.052**
Mean age of study	10 year increase	35	0.85 (0.63–1.15)	0.280	-	Not performed, 9 studies		14	0.91 (0.58–1.42)	0.639
Publication year	1 year increase	45	1.00 (0.98–1.01)	0.867	14	1.00 (0.95–1.04)	0.845	15	1.00 (0.98–1.02)	0.751

^§^number of studies.

### 3.9 Evaluation of quality of studies and risk of bias

The evaluation of quality within the eligible studies is presented in S6 and S6 Tables in S1 File for case-control and cohort studies, respectively. In case of cohort studies, the quality was mainly compromised by the ascertainment of exposure (self-administered questionnaires) and completeness of follow-up (no information). However, it has to be noted that for all studies, non-exposed individuals were selected from the same population as the exposed ones, at least one confounder was adjusted for in the analysis and follow-up was long enough in the majority of studies.

In case-control studies, hospital-based controls often compromised quality. Cases were representative in all 38 studies and were defined adequately in all but one studies. In terms of exposure, many studies contained no description of non-response rates. Nevertheless, the same method of ascertainment was uniformly guaranteed for both cases and controls in all studies; standardized, validated food frequency questionnaires were used through a structured interview for most studies.

Significant publication bias was detected *via* Egger’s test in the analysis on overall cancer risk (p<0.001), breast cancer (p = 0.013) and gastrointestinal cancer risk (p = 0.048). These results are reflected as asymmetry in the respective funnel plots (S41-S43 Figs in S1 File).

## 4. Discussion

The present systematic review and meta-analysis comprising data from 45 individual studies reveals that, overall, highest *versus* lowest olive oil consumption was associated with 31% lower cancer risk. Significant protection was noted for breast, overall gastrointestinal, upper aerodigestive and urinary tract cancer. The overall findings remained consistent when studies were further subgrouped by degree of adjustment and overall study quality, for both Mediterranean and non-Mediterranean populations, whereas they were more prominent for case-control over cohort studies.

Increasing evidence supports that olive oil constituents convey protection against the development of several types of cancer [82, 83]. The results of the present work are in agreement with relevant studies in the field. Pelucchi *et al*. in 2011 reported a summary risk ratio of breast cancer of 0.62 (95% CI: 0.44–0.88) for the highest *versus* lowest level of olive oil consumption after evaluating five case-control and one cohort study [26]. Focusing also on breast cancer risk in 2015, Xin *et al*. reported a pooled effect estimate of nine case-control and three cohort studies of 0.74 (95%CI: 0.60–0.92) [29], with the latter design giving a null association. This level of risk reduction is comparable to the one resulted from our analysis of 14 eligible studies (RR = 0.67, 95%CI: 0.52–0.86); it has to be noted though that Xin *et al*. included also articles on the use of monounsaturated fatty acids as well as olive oil combined with frying/liquid oils.

From a mechanistic point of view, our findings comply with several experimental *in vivo* and human *in vitro* studies. The favorable effect of olive oil is largely attributed to its exceptional composition, rich in monounsaturated fatty acids (mainly oleic acid) [84], squalene and phenolic compounds (simple phenols, secoiridoids and lignans) [15, 85]. Their strong anti-oxidant properties limit cellular oxidative stress and DNA damage *via* scavenging and influence crucial signaling pathways linked to carcinogenesis [86]. Regarding breast cancer, *in vitro* studies indicated that oleic acid is able to transcriptionally repress Her-2/neu overexpression and to upregulate PEA3, a transcriptional repressor of the HER2 gene [87]. It has been also observed to suppress the fatty acid synthase gene whose levels are usually increased in breast tumors [88]. In human mammary epithelial cells (MCF10A), hydroxytyrosol [89] and squalene [90] were found to reduce reactive oxygen species in the cell and protect from oxidative injury. In an experimental model of mammary cancer, a more beneficial effect was seen for mice that were fed with a diet rich in olive oil compared to a high-corn diet; additionally, the tumors were less aggressive. Underlying mechanisms involved modification of cellular membranes, signaling pathways, gene expression leading to lower proliferation, higher apoptosis and lower DNA damage [91].

Regarding gastrointestinal effects of olive oil, a plethora of preclinical evidence points to a protective role of its components [92]. In human colon cancer cells (Caco-2), extra virgin olive oil (EVOO) stimulated the expression of CNR1 gene encoding for type 1 cannabinoid receptor (CB1) and reduced proliferation. Similar increase in CB1 expression was observed in the colon of rats receiving dietary EVOO supplementation for 10 days [93]. Alu’Datt *et al*. reported that both free and lipid bound phenolic extracts of virgin olive oil exhibited antiproliferative activities against the colorectal cancer cell lines CRC1 and CRC5 [94], whereas in the studies by Hashim *et al*. the extracts limited invasion *in vitro* and metastasis *in vivo* more likely *via* modulation of integrin expression [95]. Additionally, hydroxytyrosol exerted antiproliferative effects in colon cancer cells by strong inhibition of extracellular signal-regulated kinase (ERK)1/2 phosphorylation and reduction of cyclin D1 expression [96].

Commenting on effects in the urinary tract, EVOO phenolic extract suppressed proliferation and clonogenic ability in a dose-dependent manner in human urinary bladder cancer cells (T24 and 5637) [97]. Oleuropein decreased proliferation and migration of 786-O renal cell adenocarcinoma lines [98] while hydroxytyrosol and oleuropein caused inhibition on the cancer cells of urinary bladder (T-24) [99]. The favorable antitumor effects of oleuropein and hydroxtyrosol have been extensively explored for other types of cancer such as blood, brain, hepatic, skin, cervical and thyroid [100–102].

According to the recent meta-analysis by Schwingshackl *et al*., strongest adherence to a Mediterranean diet was inversely associated with cancer mortality and risk of various cancer types; nevertheless, pooled data about the use of olive oil as a single component pointed to a non-significant effect on overall cancer risk, synthesizing a subset of relevant studies [13]. High olive oil intake may signal a healthier overall dietary pattern, interacting with other beneficial nutrients, such as those involved in the Mediterranean diet; however, the majority of the herein synthesized studies did not provide details of adherence to Mediterranean diet and correlations with intake of food groups or bioactive compounds within food groups, therefore precluding the examination of such elaborate interactions in the present meta-analysis. The portions of coexisting individual food groups and, hence, their implication to health status, are likely to differ from country to country; nevertheless, the beneficial effects in our meta-analysis spanned Mediterranean and non-Mediterranean countries.

Regarding the limitations of the present meta-analysis, between studies heterogeneity in the overall analysis was substantial but in line with previous meta-analyses [29]. Heterogeneity might be due to differences in study design, geographical region, population size, follow-up duration and other factors; in an attempt to trace its origins, we conducted a series of subgroup analyses and meta-regression analyses. Furthermore, considerable publication bias was observed, suggesting that the presence of small studies effect cannot be excluded as a factor of influence on the effect estimates; systematic reviews and meta-analyses as a research tool have also their inherent limitations, especially in the context of synthesizing smaller trials [103]. Other shortcomings pertain to the large number of case-control studies and hospital-based controls susceptible to various sources of bias, including information and selection bias. Regarding cohort studies, concerns entailed missing information on completeness of follow-up as well as the use of self-administered questionnaires for the determination of highest and the lowest category of olive intake that differed across populations. The available data, encompassing various exposure classification schemes, did not allow for dose-response evaluation; however, pooling highest vs. lowest levels of exposure is a commonly performed practice when conducting a meta-analysis.

Next, 30 out of 45 studies were limited to Mediterranean populations, where olive oil is the core of the diet, whereas none was detected in the American area, thus, compromising the generalizability of the results. Moreover, details about different types of olive oil examined (virgin olive oil, extra virgin, etc.) were not provided by the individual studies, as a rule; given that concentrations of polyphenols, antioxidants and anti-inflammatory compounds may well differ across different types of olive oil, the fact that the reporting in synthesized studies did not allow the performance of relevant subgroup analyses represents another limitation of this meta-analysis. Finally, some cancer types were not studied while others (i.e. ovarian, lung) did not generate enough eligible study arms to allow for further subgroup analyses; regarding breast cancer there was paucity of data about differential effects by menopausal status and expression of hormone receptors. Only one study [81] examined the effects of olive oil on cancer survival, leaving this field open for future exploration.

Despite the above mentioned limitations, the present work possesses a plethora of important strengths. First of all, our updated search was performed in three online databases that cover the most of biomedical literature and it was not subject to any restriction. Moreover, through strict and meticulous adherence to the PRISMA guidelines [31] as well as a careful, systematic search in reference lists (‘‘snowball” procedure) a rather impressive number of studies was achieved; 45 eligible studies in the quantitative synthesis and an overall population of approximately 1 million subjects were pooled. In contrast to previous meta-analyses, the selection procedure included articles that reported solely on olive oil consumption *per se* and not as a source of monounsaturated fatty acids or mixed with other components. Furthermore, available information was depicted on a considerable set of meaningful subanalyses and sensitivity analyses, where the favorable effects of olive oil were frequently persisted.

In conclusion, the results of this meta-analysis represent valuable evidence of the protective effects of olive oil against cancer development. Additional prospective cohort studies on various cancer types, especially in non-Mediterranean regions, as well as large randomized trials, seem desirable in order to provide further insight into the role of olive oil in preventing cancer.

## Supporting information

S1 File (RAR)

## Abbreviations

CB1: cannabinoid receptor type 1 (CB1)
CI: confidence interval
d: day
EVOO: extra virgin olive oil
g: grams
gpd: grams per day
gpw: grams per week
HER2: human epidermal growth factor receptor 2
I^2^: inconsistency index
lpm: liters per month
NC: not calculable
NOS: Newcastle-Ottawa score
PEA3: polyomavirus enhancer activator 3
PRISMA: Preferred Reporting Items for Systematic Reviews and Meta-Analyses guidelines
RR: relative risk
Qn: quintile
Qr: quartile
spw: servings per week
T: tertile
tabspy: tablespoon per year
tpm: times per month
tpw: times per week
